# Device quantization policy in variation-aware in-memory computing design

**DOI:** 10.1038/s41598-021-04159-x

**Published:** 2022-01-07

**Authors:** Chih-Cheng Chang, Shao-Tzu Li, Tong-Lin Pan, Chia-Ming Tsai, I-Ting Wang, Tian-Sheuan Chang, Tuo-Hung Hou

**Affiliations:** Department of Electronics Engineering and Institute of Electronics, National Yang Ming Chiao Tung University, Hsinchu, 300 Taiwan

**Keywords:** Electrical and electronic engineering, Information technology

## Abstract

Device quantization of in-memory computing (IMC) that considers the non-negligible variation and finite dynamic range of practical memory technology is investigated, aiming for quantitatively co-optimizing system performance on accuracy, power, and area. Architecture- and algorithm-level solutions are taken into consideration. Weight-separate mapping, VGG-like algorithm, multiple cells per weight, and fine-tuning of the classifier layer are effective for suppressing inference accuracy loss due to variation and allow for the lowest possible weight precision to improve area and energy efficiency. Higher priority should be given to developing low-conductance and low-variability memory devices that are essential for energy and area-efficiency IMC whereas low bit precision (< 3b) and memory window (< 10) are less concerned.

## Introduction

Deep neural networks (DNNs) have achieved numerous remarkable breakthroughs in applications such as pattern recognition, speech recognition, object detection, etc. However, traditional processor-centric von-Neumann architectures are limited in energy efficiency for computing contemporary DNNs with rapidly increased data, model size, and computational load. Data-centric in-memory computing (IMC) is regarded as a strong contender among various post von-Neumann architectures for reducing data movement between the computing unit and memories for accelerating DNNs^1,2^. Furthermore, quantized neural networks (QNNs) that truncate weights and activations of DNNs have been proposed to further improve the hardware efficiency^3^. Compared with the generic DNNs using floating-point weights and activations, QNNs demonstrate not only substantial speedup but also a tremendous reduction in chip area and power^4^. These are accomplished with nearly no or minor accuracy degradation in the inference tasks of complex CIFAR-10 or ImageNet data^3^. While the emergence of QNNs opens up the opportunity of implementing IMC using emerging non-volatile memory (NVM), the practical implementation is largely impeded by the imperfect memory characteristics, in particular, only a limited number of quantized memory (weight) states is available at the presence of intrinsic device variation. The intrinsic device variation of emerging NVM such as PCM^5^, RRAM^6^, MRAM^7^, leads to a significant degradation in inference accuracy.

Number of studies have investigated these critical issues with the focus on the impact of the DNN inference accuracy^7–9^. NV-BNN focused on the impact of binary weight variation on inference accuracy, but the considerations on multi-bit weights and the impact on energy and area efficiency are lacking^7^. Yang et al. evaluated the impact of DNN inference accuracy based on various state-of-the-art DNNs^8^. The major focus was on the impact of noise of input and weight while considering hardware efficiency. Yan et al. discussed the energy/area efficiency and the impact of device non-ideality on inference accuracy separately but not simultanously^9^. Therefore, a comprehensive and quantitative study that links the critical device-level specs, namely quantization policy, memory dynamic range, and variability with those system-level specs, namely power, performance (accuracy), and area (PPA), is still lacking. This paper intends to provide a useful guide on NVM technology choice for quantized weights considering the variation-aware IMC PPA co-optimization. Different from the previous works, our evaluation takes into account practical IMC design options at the architecture, algorithm, device, and circuit levels. First, we compare the inherent immunity against device variation among different IMC mapping schemes. Then, the dependence of variation immunity on DNN algorithms, quantization methods, weight precision, and device dynamic range are discussed. To further improve the immunity against variation, the strategies of using multiple cells to represent a higher-precision weight and fine-tuning last fully-connect classifier layer in the network are detailed. Finally, taking into account other circuit-level constraints, such as limited current summing capability and peripheral circuit overhead, the energy and area efficiency of variation-aware IMC designs are compared to provide a useful guideline for future IMC PPA co-optimization.

## Results

### Background

In a neural network, a set of weight matrix *W*_M×N_ is assigned to an input vector *I*_M×1_. During the feedforward calculation, the vector–matrix multiplication (VMM) between the input vector and the weight matrix is performed to generate an output vector *O*_1×N_. In the IMC architecture, the weight matrix *W*_M×N_ is represented using cell conductance in an orthogonal memory array (*G*_M×N_). The VMM is performed by applying the voltage input vector *v*_M×1_ to the array and measuring the current output vector *i*_1×N_ by summing currents flowing through all cells in every column. Each memory cell could be regarded as a multiply-accumulate (MAC) unit. Thus, the high-density array allows extremely high parallelism in computing. The IMC-based VMM accelerates general matrix multiply (GEMM), which counts for over 70% of DNN computational load^10^, by using stationary weights in the memory array.

The weights in DNNs algorithms are signed values. It is important to allow negative weights for capturing the inhibitory effects of features^11^. To implement signed weights using only positive conductance of memory devices, an appropriate mapping scheme is required. Depending on the choice of the activation function, the activation values and also the input values in neural networks are either with negative values (e.g., hard tanh) or without negative values (e.g., ReLU). This also affects the choice of DNN-to-IMC mapping schemes. Besides, compared to software-based DNNs, IMC hardware tends to use lower precision for data representation and computing to achieve better energy and area efficiency. In the following subsection, we will introduce different kinds of DNN-to-IMC mapping schemes and how to implement quantized weights using emerging NVMs.

#### DNN-to-IMC mapping

In this work, we consider a one-transistor one-resistor (1T1R) memory array for illustrating various DNN-to-IMC mapping schemes. Each memory unit cell in the 1T1R array consists of a selection transistor and a two-terminal memory device with changeable resistance. One of the terminals of the memory device is connected to the drain of the transistor through a back-end-of-line via. The word line (WL), bit line (BL), and source line (SL) are connected to the transistor gate, the other terminal of the memory device, and the transistor source, respectively. The WLs and SLs are arranged orthogonally to the BLs.

**Figure 1:**
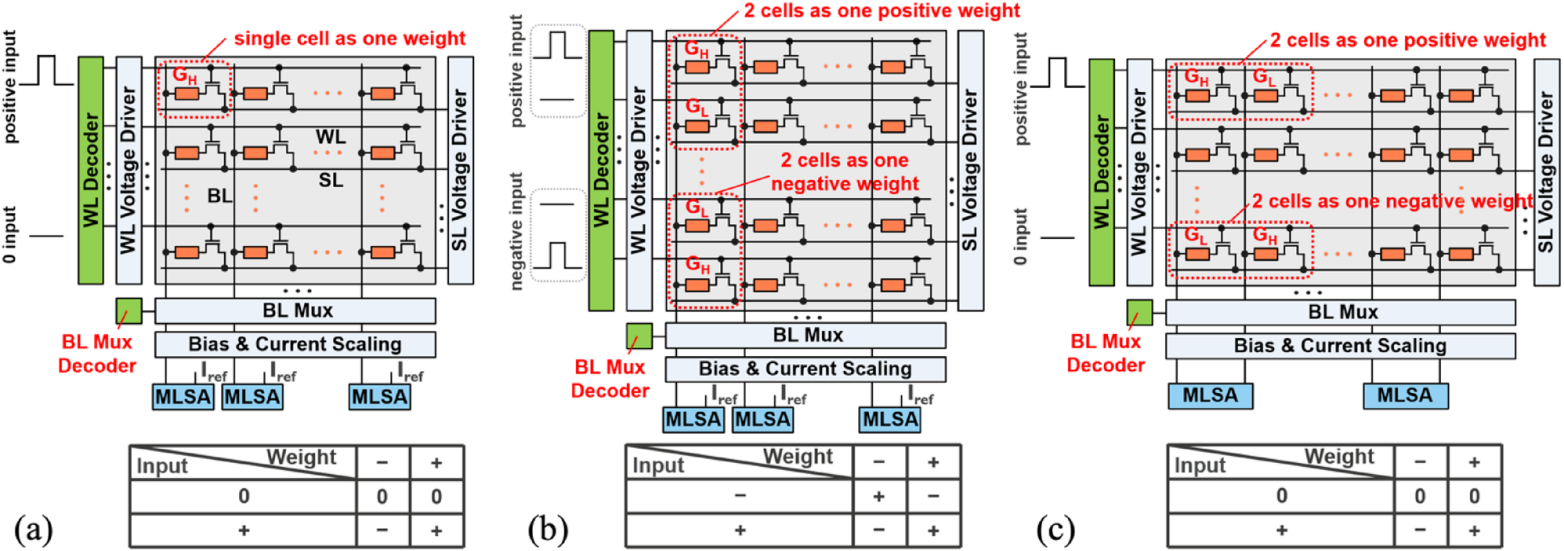
DNN-IMC mapping schemes. (**a**) Naïve IMC with ± weight and + input. (**b**) Complementary IMC with ± weight and ± input. (**c**) Weight-separate IMC with ± weight and + input.

Three commonly used mapping schemes are considered in this work. The naïve IMC (N-IMC) scheme (Fig. 1a) uses a single memory unit cell and a single WL to represent a positive/negative weight (± *w*) and positive input (+ IN), respectively^2,12–14^. A constant voltage bias is clamped between BLs and SLs. When the input is zero, WL is inactivated and no current generates from the cells on the selected WL. When the input is high, WL is activated and the summing current flowing from the cells on the same BL is sensed. To represent both the sign and value of weights using a single cell, an additional reference current is required to compare with the BL current via a sensing amplifier (SA) or an analog-to-digital converter (ADC) to obtain the final MAC result. The complementary IMC (C-IMC) (Fig. 1b) uses two adjacent memory cells on the same BL with complementary conductance to represent both ± *w* and two WLs with a set of complementary inputs to represent ± IN^15,16^. The weight-separate IMC (WS-IMC) (Fig. 1c) uses the conductance difference of two adjacent memory cells on the same WL with complementary conductance to represent the sign and value of weight. Two BL currents are directly compared with no need for additional reference^17–19^. Similar to N-IMC, WS-IMC uses a single WL to present only + IN. These three different schemes have both pros and cons. N-IMC is the most compact. C-IMC with ± IN is compatible with most software algorithms. WS-IMC requires no external reference. In QNNs based on all three schemes, the quantized inputs could be encoded using multi-cycle binary pulses applied to the WL (transistor gate) without using high-precision digital-to-analog converters (DACs). An analog current adder is used to combine MAC results in multiple cycles to obtain the final activation values through ADCs^20^. Note that the 1-bit input/activation by using the simple SA is first assumed in our later discussion to avoid the high energy and area overheads in ADCs. In “Variation-aware PPA co-optimization” section, we will further discuss the impact of high-precision input/activation on the IMC design.

#### Quantized weight

**Figure 2:**
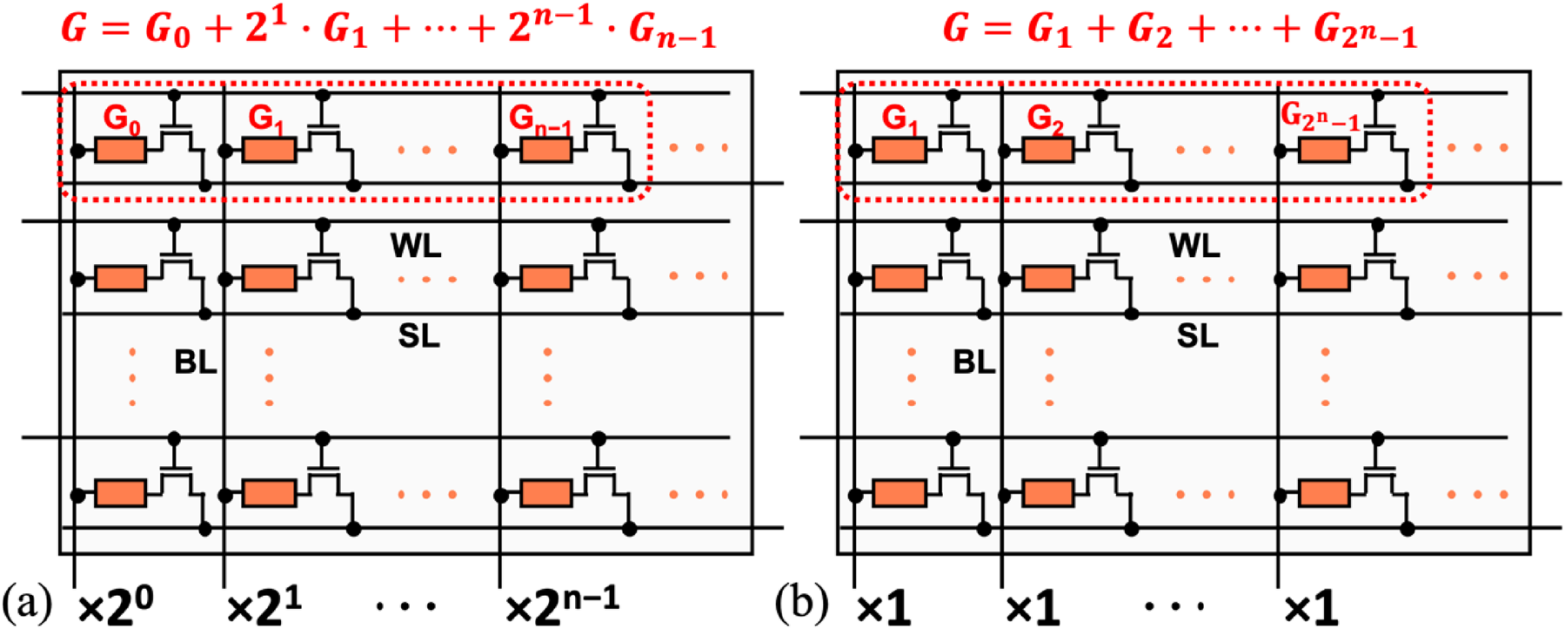
Multiple cells per weight schemes to represent a higher precision weight. (**a**) Digital MLC using *N* 1-bit cells on the same WL to represent an *N*-bit weight. (**b**) Analog MLC using 2^*N*^*-1* 1-bit cells on the same WL to represent an *N*-bit weight.

To implement quantized weight in QNNs, the multi-level-cell (MLC) memory technology that provides sufficient precision is the most straightforward choice, which we refer to straightforward MLC (S-MLC)^12,15–17,19^. Besides, multiple memory cells where each has a lower precision could be used to implement a weight with higher precision. This allows using even binary (1-bit) memory technology to realize versatile QNNs at the expense of area. Two schemes, which we refer to digital MLC (D-MLC)^13,18^ and analog MLC (A-MLC)^14^, are possible (Fig. 2a,b). The former sums the BL currents of the most-significant-bit (MSB) cell to the less-significant-bit (LSB) cell using the power of two weighting while the latter uses the unit weighting. For example, the numbers of cells per weight are *N* and 2^*N*^ − 1, respectively, for an *N*-bit weight in the N-IMC mapping by using a 1-bit memory cell.

Table 1 summarizes the DNN-to-IMC mapping schemes and quantized weight implementation methods recently proposed in the literature. Both volatile SRAM and non-volatile RRAM and PCM are popular choices for IMC. All three DNN-to-IMC mapping schemes, N-IMC, C-IMC, and WS-IMC, have been investigated in different studies. S-MLC and its most primitive form by using only binary memory cells are prevalent while D-MLC and A-MLC are used for implementing high-precision weights using low-precision cells. However, how the inherent variation of memory states influences the optimal choice among various IMC architectures has yet to be investigated comprehensively.

**Table 1:** Summary of various DNN-to-IMC mapping schemes and quantized weight implementation methods proposed in the literature.

	HP lab^12^	ISAAC^13^	ASU^14^	XNOR-SRAM^15^	XNOR-RRAM^16^	IBM^17^	PRIME^18^	NTHU^19^
Mapping	N-IMC	N-IMC	N-IMC	C-IMC	C-IMC	WS-IMC	WS-IMC	WS-IMC
Weight	S-MLC	D-MLC	A-MLC	S-MLC	S-MLC	S-MLC	D-MLC	S-MLC
Device	RRAM	N/A	RRAM	SRAM	RRAM	PCM	RRAM	RRAM
Cell precision	5-bit	2-bit	6-bit	1-bit	1-bit	5-bit	4-bit	1-bit
Weight precision	5-bit	16-bit	6-bit	1-bit	1-bit	6-bit	8-bit	1-bit
Activation precision	4-bit	8-bit	N/A	1-bit	1-bit	8-bit	6-bit	1-bit

### Finite quantized memory state

**Figure 3:**
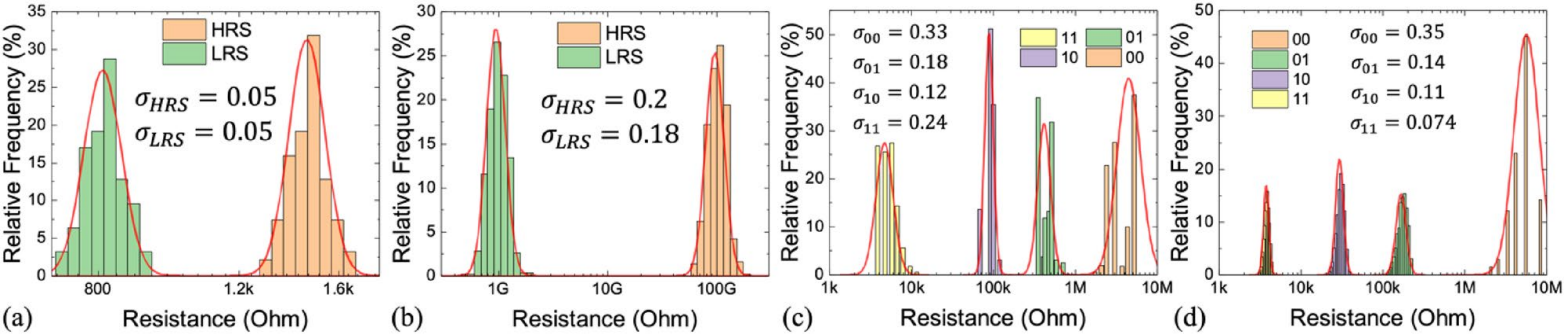
DtD conductance (G) variation of memory states fit the log-normal distribution in (**a**) STT-MRAM, (**b**) FTJ^23^, (**c**) MLC PCM^5^, (**d**) MLC RRAM^6^. The standard deviations (*σ*) do not change significantly in the same device except for the lowest G (00) state in MLC PCM and RRAM. The higher variation of the (00) state has less impact on IMC accuracy. Thus, we adopted a constant *σ* in the log-normal distribution as the variation model.

Although rich literature has discussed various process innovation^21^ or closed/open-loop programming schemes^22^ to increase the number of quantized memory states, the ultimate number of quantization levels in a memory device is determined by the dynamic range, *e.g.* conductance ratio (*G*_H_/*G*_L_) in a resistance-based memory, and the device-to-device (DtD) variation. The DtD variation limits how accurate weight placement is. We found the standard deviations (*σ*) in the log-normal conductance distribution does not change significantly with the conductance value in the same device. Figure 3 shows the statistical histograms for binary MRAM, ferroelectric tunnel junction (FTJ)^23^, MLC PCM^5^, and RRAM^6^, respectively. G-independent *σ* is used as the device variation model in the following discussion for simplicity. The influence of G-dependent *σ* is further discussed in Fig. S1 (Supporting Information).

**Figure 4:**
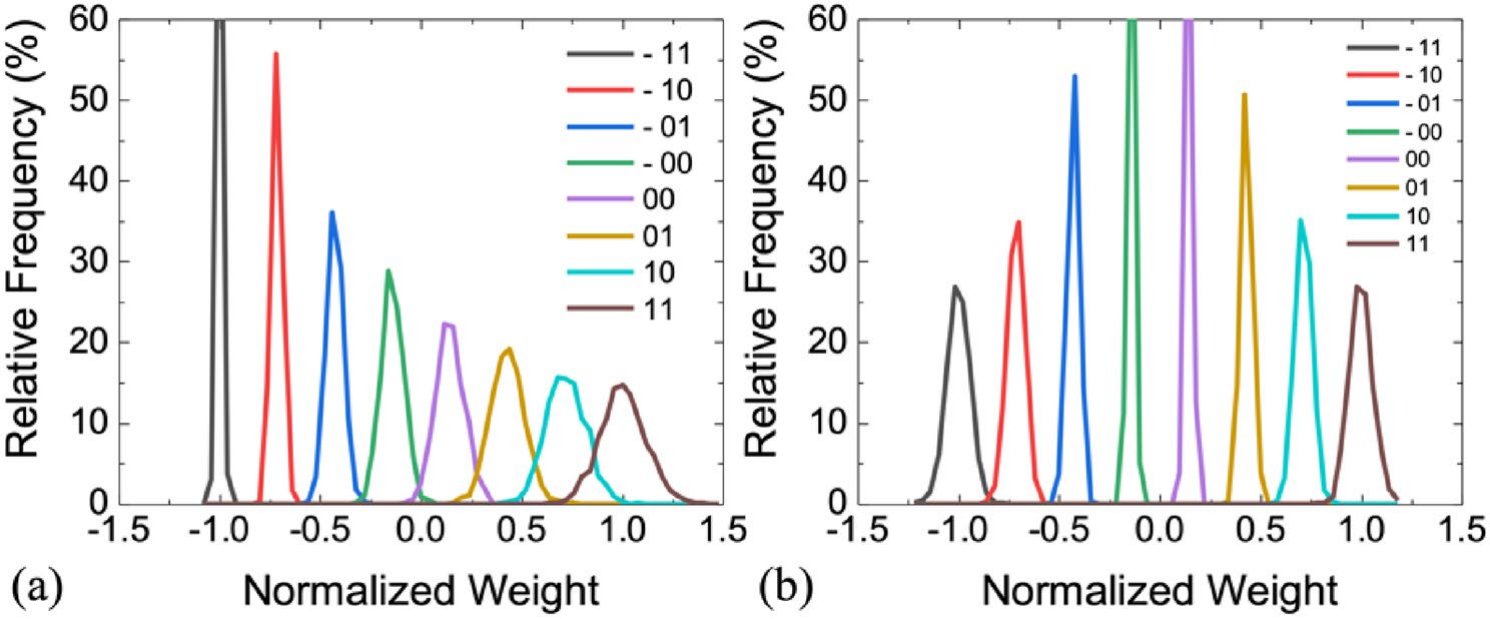
Weight distribution of 3-bit linear quantization (S-MLC, *G*_H_/*G*_L_ = 10, *σ* = 0.05). (**a**) N-IMC mapping. Variation is higher at + *w* with high conductance in the linear scale. (**b**) WS-IMC mapping. Variation is symmetric at + *w* and − *w*.

Figure 4 shows an example of the weight distribution of 3-bit linear quantization (Lin-Q) in the N-IMC and WS-IMC mapping scheme by using the S-MLC weight. Because of the constant *σ* in the log-normal scale, the distribution of the *G*_H_ states for representing + *w* appears broader compared with the *G*_L_ states for representing − *w* in the linear scale for the N-IMC scheme (Fig. 4a)^6^. While the weight distribution is asymmetric in N-IMC, it is symmetric for ± *w* in WS-IMC (Fig. 4b). This is because the same conductance difference of two adjacent cells is used to represent the value of the signed weights. Although C-IMC utilizes two cells in the same column to represent one weight, only one cell between the two is accessed at a time because of the complementary inputs applied to the transistor gate terminal of the 1T1R cell. Therefore, both the weights of C-IMC and N-IMC schemes are based on the difference between the device conductance of one cell and the reference. So the weight distribution of C-IMC is identical to that of N-IMC.

### Quantization policy for accurate inference

**Figure 5:**
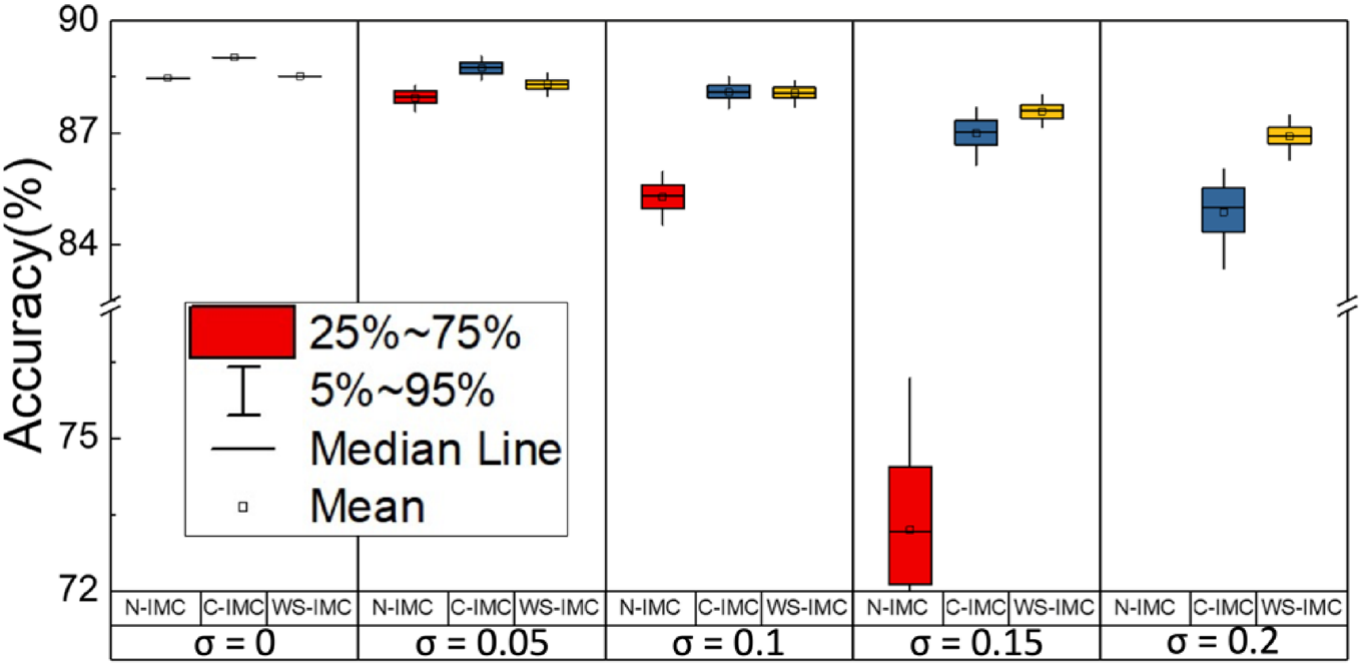
Influence of IMC mapping schemes. CIFAR-10 inference accuracy using VGG-9, 1-bit weight, 1-bit activation, *G*_H_/*G*_L_ = 100 are compared. The statistical effect of conductance variation was evaluated using the Monte Carlo simulation of at least 200 times.

All three schemes discussed in “DNN-to-IMC mapping” section could achieve comparable accuracy after appropriately training the models when the device variation is negligible. However, their immunity against device variation differs substantially. Figure 5 shows the inference accuracy of VGG-9 DNNs for CIFAR-10 classification with different levels of variability. The weight placement considering the log-normal conductance distribution and G-independent *σ* was evaluated using the Monte Carlo simulation of at least 200 times. The distribution of these 200 data points was plotted in Fig. 5. As *σ* increases, the inference accuracy degrades. N-IMC is the worst mainly due to the error accumulation from + *w* with broader distributions of *G*_L_ states as compared with − *w*, as apparent in Fig. 4a. C-IMC shows improvement on inference accuracy compared with N-IMC because of the error cancellation effect originated from the complementary input. Note that the generation of complementary inputs requires additional hardware cost. WS-IMC is the most superior against variation among three because of the error cancellation from the symmetric and tighter ± w distribution (Fig. 4b) that is constituted by two cells but not one, and it requires no complementary input. More detailed comparison between these three schemes with different G_H_/G_L_ could be found in Fig. S2 (Supporting Information). For the rest of this paper, only the median values of inference accuracy in the Monte Carlo simulation and the WS-IMC mapping scheme are discussed for simplicity.

Besides DNN-to-IMC mapping schemes, different design considerations at the algorithm and device levels also affect the inference accuracy in the presence of device variation. In the following subsections, we will further discuss the impact of choices of networks and datasets, quantization function, weight (conductance) precision, and dynamic range on inference accuracy.

#### Network choice

Variation immunity is known to be sensitive to the choice of DNN algorithms^8^. Both VGG-16 and ResNet-18 are compared by using a more complex Tiny ImageNet dataset, as shown in Fig. S3 (Supporting Information). The compact ResNet-18 with 11.4 M parameters deteriorates the variation immunity compared with the VGG-16 with 134.7 M parameters^8^. For example, for a device with 1-bit weight, G_H_/G_L_ = 100, and *σ* = 0.1, the accuracy for ResNet-18 and VGG-16 are 39.82% and 47.57%, respectively. Therefore, only the VGG-DNNs with better immunity against variation are further evaluated below.

#### Lin-Q vs. Log-Q

**Figure 6:**
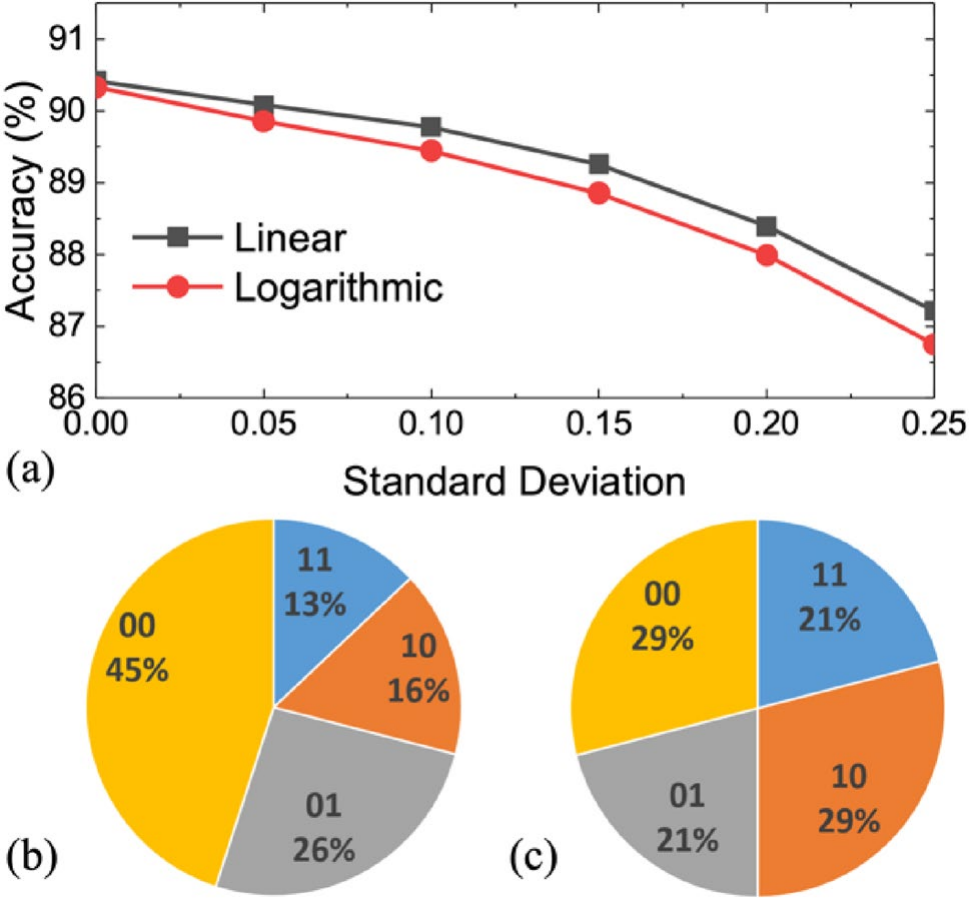
(**a**) CIFAR-10 inference accuracy comparison using linear and logarithmic quantization with variations. 3-bit weight, *G*_H_/*G*_L_ = 100, and WS-IMC mapping are used. The distribution of different quantized weight levels using (**b**) linear and (**c**) logarithmic quantization.

Logarithmic quantization (Log-Q) is favored for multi-bit memory storage because a larger memory sensing margin is possible by avoiding overlapping of tailed bits between levels. Previous studies also attempted to use Log-Q for the weights of DNNs^24^. Our simulation shows that after appropriate training both Log-Q and Lin-Q achieve comparable accuracy in the ideal quantization case without variation. However, Lin-Q shows more robust immunity against variation than Log-Q, as shown in Fig. 6. This is explained by their different weight distributions. In Log-Q, more weights are located at ± 11 states which have a wider weight distribution. Therefore, the larger sensing margin between levels in Log-Q does not necessarily guarantee better immunity against variation. Only Lin-Q is further discussed in this study.

#### Weight quantization precision and dynamic range

**Figure 7:**
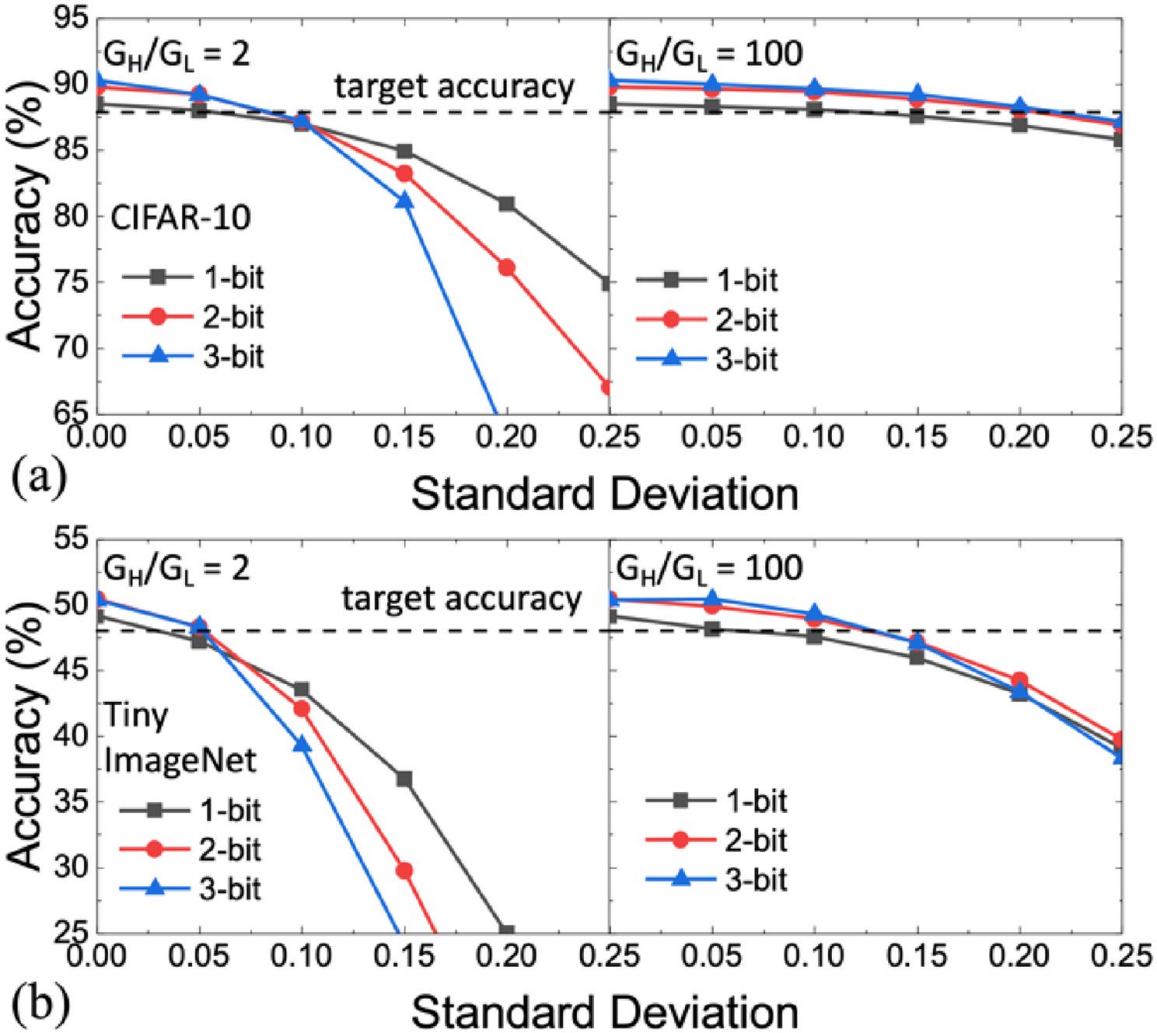
Impact of linear quantization policy considering weight precision and *G*_H_/*G*_L_. (**a**) CIFAR-10 inference (VGG-9) accuracy using *G*_H_/*G*_L_ = 2 and *G*_H_/*G*_L_ = 100. (**b**) Tiny ImageNet inference (VGG-16) using *G*_H_/*G*_L_ = 2 and *G*_H_/*G*_L_ = 100. 1-bit activation is assumed. Higher weight precision improves the baseline accuracy but is more susceptible to variation. Enlarging *G*_H_/*G*_L_ improves immunity against variation.

**Figure 8:**
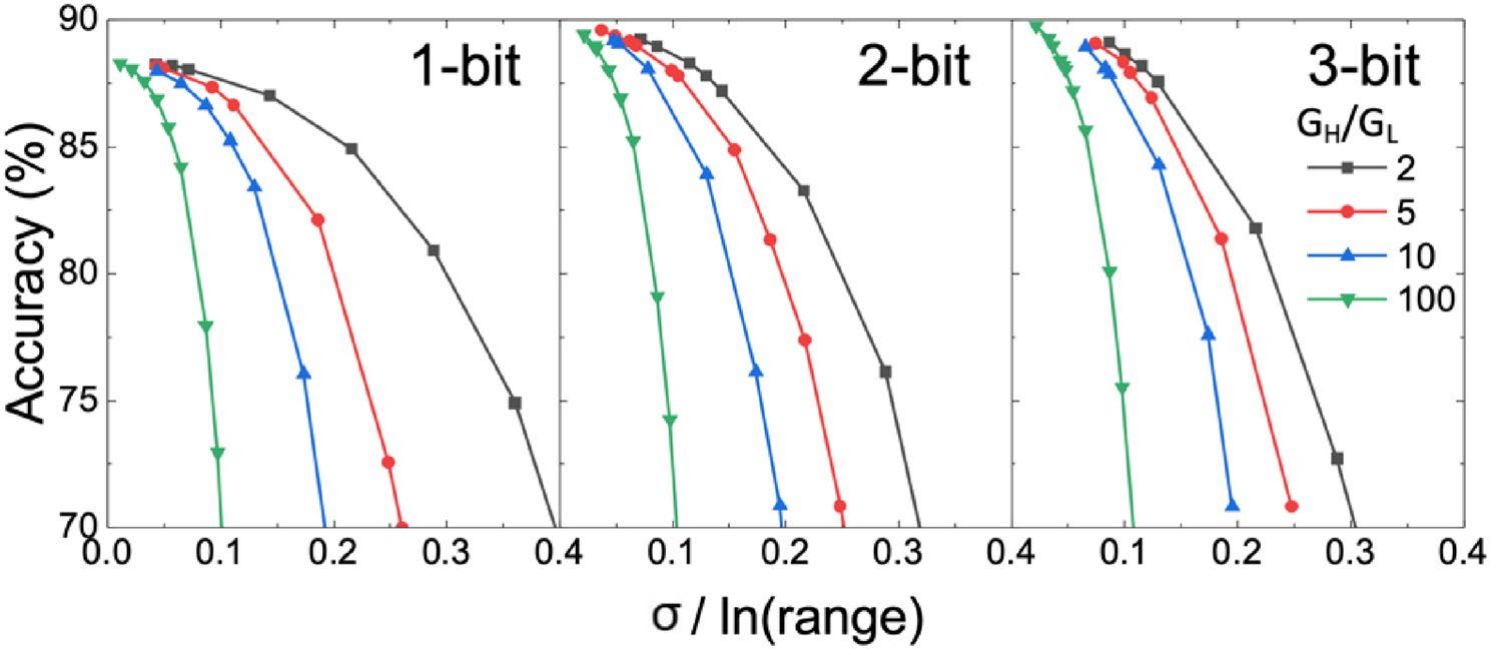
Normalized *σ* vs. accuracy and the impact of dynamic range and weight precision. *σ *is normalized to ln(*G*_H_/*G*_L_) considering the log-normal distribution of variation in memory states.

The immunity to variation is further investigated in the models with different weight precision from one to three bits in Fig. 7. The focus on the lower weight precision considers only inference but not training applications and also the reality of using the existing memory technology for realizing MLC. Here we also take into account the influence of conductance dynamic range *G*_H_/*G*_L_. The major conclusions are: (1) Although the high weight precision improves the baseline accuracy in the ideal case, it is more susceptible to variation. The accuracy could be even worse than using low weight precision if the variation is substantial. For the first order, this effect could be explained as follows: For a higher weight precision, a larger number of weight states are placed within a given dynamic range. The margin between each state becomes less compared with the case with a lower weight precision. The same degree of variation (same σ) would distort the pre-trained model more significantly and result in more severe accuracy degradation. (2) Enlarging the dynamic range is beneficial to the variation immunity for a given *σ*. However, at the same normalized *σ*, i.e. *σ*/ln(G_H_/G_L_), a smaller dynamic range with smaller device variation is favorable than a larger dynamic range with larger device variation, as shown in Fig. 8. The result suggests that a low absolute value of *σ* is still critical for the model accuracy. Higher priority should be given to suppressing variation rather than enlarging the dynamic range. (3) A more complicated dataset (Tiny ImageNet vs. CIFAR-10) is more susceptible to variation since the model itself also becomes more complicated, but it does not change the general trends aforementioned.

#### Variation-aware accurate DNN

**Figure 9:**
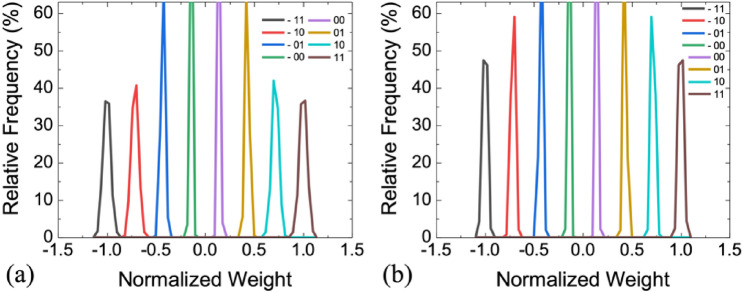
Weight distribution of 3-bit linear quantization (*G*_H_/*G*_L_ = 10, σ  = 0.05 for 1-bit cell) using (**a**) the WS-IMC + D-MLC mapping scheme and (**b**) the WS-IMC + A-MLC mapping scheme, showing tighter distributions than S-MLC in Fig. 4b.

Two approaches are further evaluated to improve the immunity against variation. First, the D-MLC and A-MLC weights, as introduced in “Quantized weight” section, are more robust against variation than the S-MLC weight. Figure 9 shows an example of the weight distribution of 3-bit linear quantization in the WS-IMC mapping scheme by using the D-MLC and A-MLC weight, respectively. The D-MLC and A-MLC weights consist of three and seven binary (1-b) memory cells, respectively, with the identical *G*_H_/*G*_L_ and σ as those in Fig. 4b for the S-MLC weight. Because more cells are used to represent a weight for D-MLC and A-MLC, the “effective” *σ* for a given quantized weight precision is reduced due to the averaging effect from the law of large numbers. Second, the inference accuracy degradation could be partially recovered by fine-tuning the last fully-connect classifier layer in the network^7^. The last classifier layer is a full-precision layer that could be easily implemented using the conventional digital circuits. After placing weights in all IMC layers, the weights in the digital classifier layer is retrained with all weights in the IMC layers fixed. The computing efforts for retraining only the classifier layer is relatively small. The retrain speed is fast because it requires only a subset of data instead of a complete training epoch^7^.

Tables 2 and 3 summarize the maximum tolerable variation for CIFAR-10 and Tiny ImageNet, respectively, by using different quantization policies, including quantization precision, dynamic range, and weight implementation scheme. The pre-defined target accuracy for CIFAR-10 using VGG-9 and Tiny ImageNet using VGG-16 are 88% and 48%, respectively. To achieve the proposed targets with relatively high accuracy, higher weight precision (2/3b vs. 1b) is beneficial because it increases the baseline accuracy, thus allowing more variation tolerance. Enlarging *G*_H_/*G*_L_ is also beneficial. Among the three weight implementation schemes, A-MLC shows the best variation tolerance due to its smallest “effective” *σ* obtained from multiple devices. Furthermore, the fine-tuning technique is extremely useful for boosting variation tolerance. So it should be applied whenever possible if device-level solutions for reducing *σ* are not available.

**Table 2:** Tolerable variation for CIFAR-10 (VGG-9 @ 88% acc.) with and without fine-tuning (FT).

Dynamic range	Weight precision	Weight implemented scheme	Tolerable STD w/o FT	Tolerable STD w/ FT
G_H_/G_L_ = 2	1-bit	S-MLC	0.05	0.07
	2-bit	S-MLC	0.08	0.11
		D-MLC	0.11	0.15
		A-MLC	0.14	0.19
	3-bit	S-MLC	0.08	0.11
		D-MLC	0.12	0.27
		A-MLC	0.2	0.28
G_H_/G_L_ = 100	1-bit	S-MLC	0.1	0.17
	2-bit	S-MLC	0.2	0.29
		D-MLC	0.24	0.37
		A-MLC	0.26	0.46
	3-bit	S-MLC	0.22	0.3
		D-MLC	0.27	0.66
		A-MLC	0.33	0.66

**Table 3:** Tolerable variation for Tiny ImageNet (VGG-16 @ 48% acc.) with and without fine-tuning (FT).

Dynamic range	Weight Precision	Weight implemented scheme	Tolerable STD w/o FT	Tolerable STD w/ FT
G_H_/G_L_ = 2	1-bit	S-MLC	0.03	0.08
	2-bit	S-MLC	0.05	0.1
		D-MLC	0.06	0.12
		A-MLC	0.08	0.16
	3-bit	S-MLC	0.05	0.1
		D-MLC	0.08	0.16
		A-MLC	0.13	0.27
G_H_/G_L_ = 100	1-bit	S-MLC	0.08	0.27
	2-bit	S-MLC	0.13	0.24
		D-MLC	0.14	0.27
		A-MLC	0.18	0.39
	3-bit	S-MLC	0.13	0.25
		D-MLC	0.17	0.37
		A-MLC	0.22	0.61

### Variation-aware PPA co-optimization

**Figure 10:**
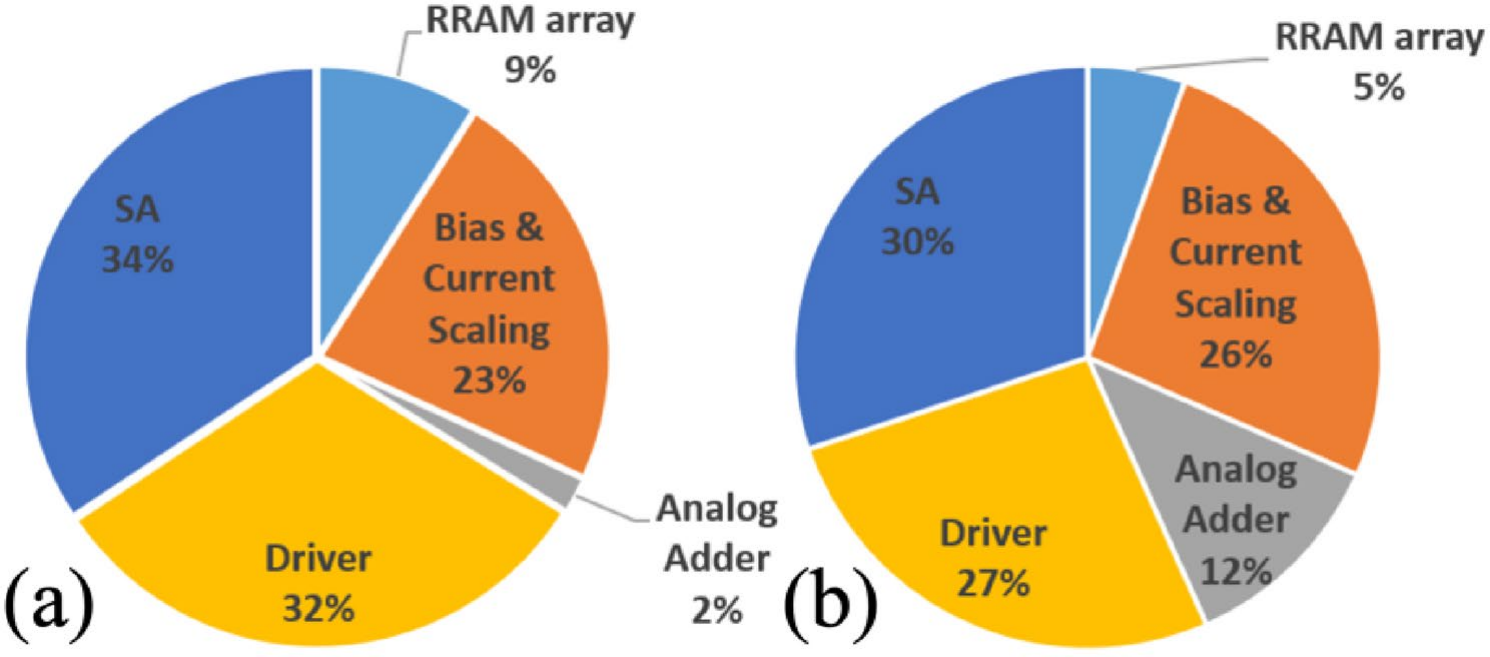
(**a**) Area and (**b**) power breakdown of a 40 nm 256 × 256 1T1R IMC macro.

Some of the strategies for improving IMC variation immunity accompany penalties in power and area. A larger *G*_H_/*G*_L_ implies that the *G*_H_ cell is forced to operate in a higher current regime. Here we assume the minimum of *G*_L_ is finite and limited by the leakage in given memory technology. Previous studies have shown that a high BL current creates substantial voltage drop on the parasitic line resistance and results in inaccurate MAC results. Partitioning a large array with high BL currents to smaller ones is necessary to guarantee the model accuracy^23^. The higher *G*_H_ thus restricts the attainable maximum sub-array size because of the excessively large accumulated current on BLs. The increased BL current with higher *G*_H_ deteriorates energy efficiency while the smaller sub-arrays with higher *G*_H_ deteriorates area efficiency due to higher peripheral circuit overhead. D-MLC and A-MLC by using more memory cells also increase the area and energy consumption of IMC. Therefore, the variation tolerance should be carefully traded off with efficient hardware design. To fairly evaluate the PPA of IMC with different device specifications, we completed a reference design based on the foundry 40-nm CMOS technology with a 256 × 256 1T1R RRAM array macro. The major circuit blocks in the macro are similar to the illustration shown in Fig. 1. We assume a hypothetical memory with a fixed low conductance state (*G*_L_ = 0.5 μS) and G_H_/G_L_ = 10, 1-bit input/activation, and the WS-IMC/S-MLC mapping. The IMC sub-array size is limited by the maximum allowed BL current of 300 μA through current-mode sensing. Fig. 10 shows a simulated power and area breakdown of the IMC macro, which includes bias clamping and current scaling circuits, current-mode SAs, analog adders to accumulate the partial sums from different sub-arrays, and driver circuits for WL/BL/SL. Other IMC designs using different *G*_H_/*G*_L_ ratios (assuming G_L_ is fixed), D-MLC/A-MLC weights, multi-cycle inputs, and multibit ADCs are then extrapolated using the reference design.

**Figure 11:**
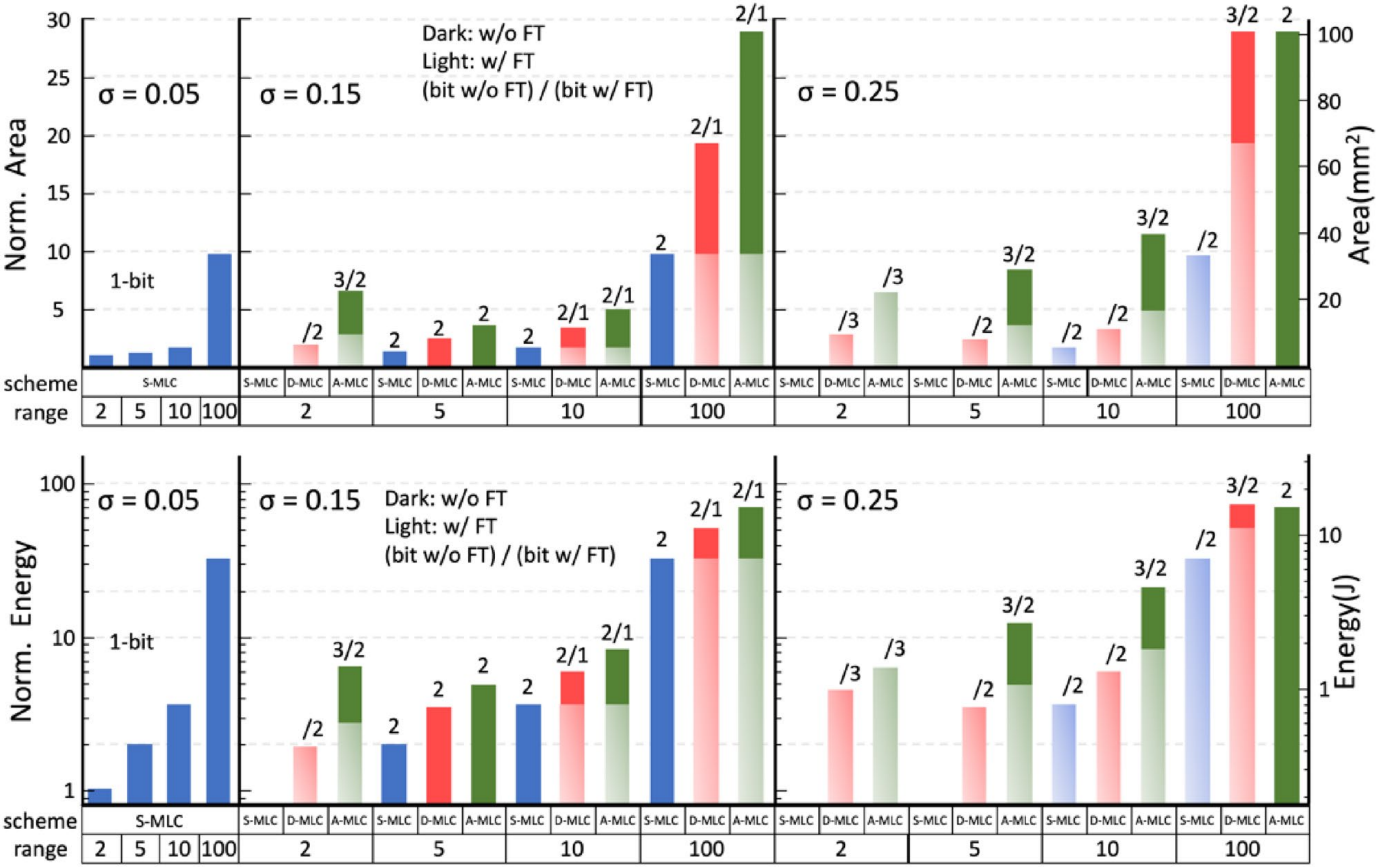
Area and energy estimation of feasible IMC designs that guarantees CIFAR-10 inference (VGG-9) with at least 88% accuracy (see Table 2). Designs considering different standard deviation of conductance distribution, *G*_H_/*G*_L_ ratio, and S-MLC/D-MLC/A-MLC scheme are compared, and the lowest possible weight precision is used to simplify the hardware implementation. 1-bit activation is assumed. Dark and light colors indicate the estimation w/o and w/ considering fine-tuning. The fine-tuning results are shown only when fine- tuning helps to reduce the weight precision required. The lowest weight precision required is also indicated.

**Figure 12:**
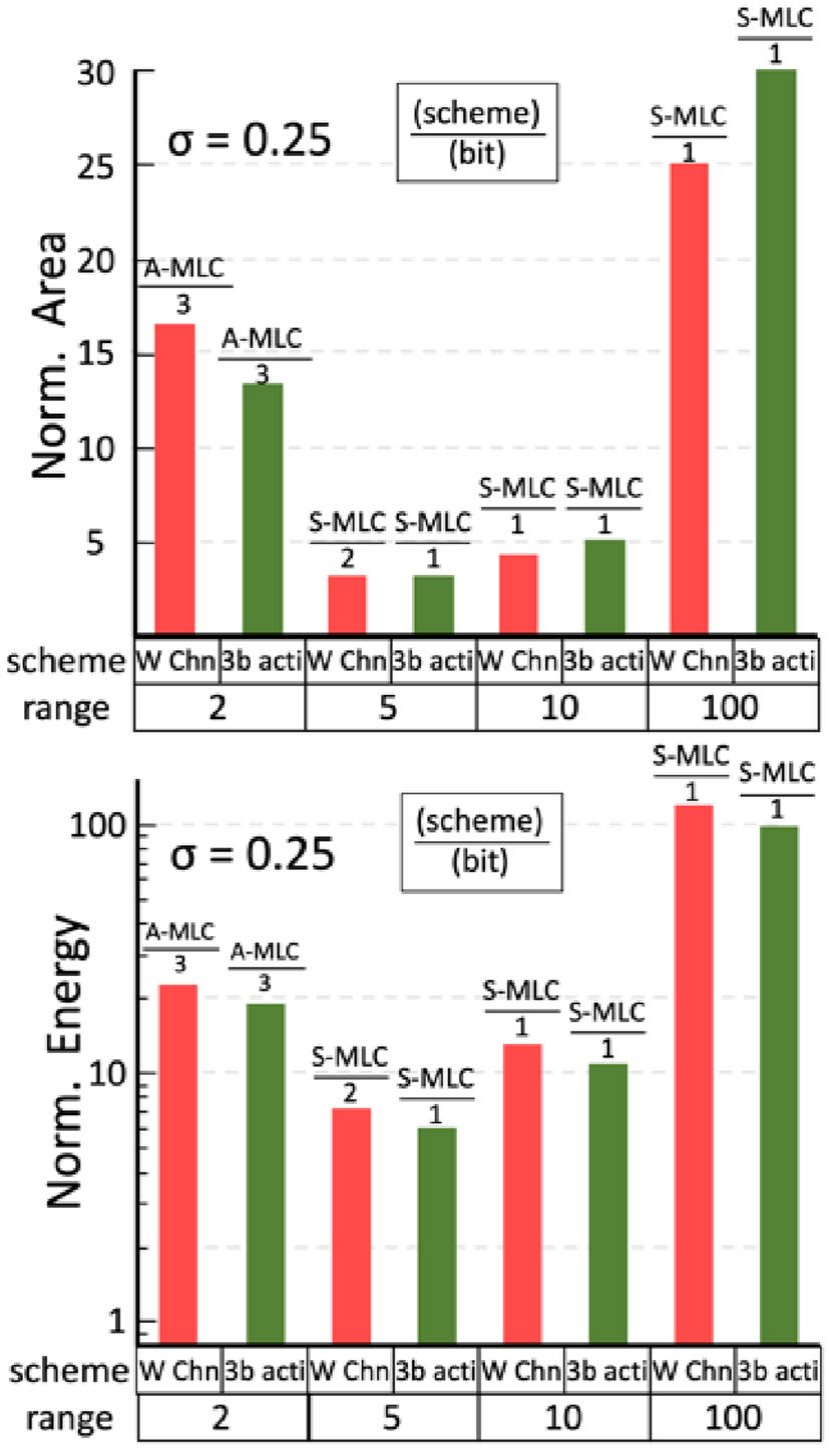
Area and energy estimation of IMC designs using the same criteria as Fig. 11 but with either wider channel or 3-bit activation. The improvements only exist in specific conditions with high *σ*.

The area and energy of feasible designs for VGG-9 that satisfy the pre-defined accuracy target (e.g. Table 2) are compared in Fig. 11. The trends for VGG-16 are similar and not shown here. The lowest weight precision is used whenever possible to relax device requirements and system overhead. The energy is estimated by the total energy consumption of 10,000 CIFAR-10 inferences. We summarize the strategies on the PPA co-optimization as follows: (1) For a *low-variation* device (*σ* = 0.05), a *binary cell* with low *G*_H_/*G*_L_ allows the highest area and energy efficiency. (2) For a *moderate-variation* device (*σ* = 0.15), *S-MLC* with moderate *G*_H_/*G*_L_ (< 10) achieves better efficiency. (3) For a *high-variation* device (*σ* = 0.25), using S-MLC becomes challenging unless the fine-tuning is considered. Using *D-MLC/A-MLC* with moderate *G*_H_/*G*_L_ is practical alternatives to maintain accuracy at a reasonable cost of energy and area. Other variation-aware strategies that could affect the PPA of IMC include using a higher (3-bit) input/activation precision and more channels (2 times more) in a wider VGG network. The complete area and energy estimations of these variation-aware strategies are shown in Fig. S4 (Supporting Information). Only those most efficient schemes using the lowest possible bit precision for satisfying the target accuracy are plotted in Fig. 12 for each dynamic range. Our evaluations show that the substantial penalties on area and energy restrict these strategies only competitive in specific conditions, especially when *σ* is large.

## Conclusion

In this paper, we provided an end-to-end discussion for the impact of intrinsic device variation on the system PPA co-optimization. We considered critical device-level constrains, such as limited quantization precision and memory dynamic range, circuit-level constraints, such as limited current summing capability and peripheral circuit overhead, architecture-/algorithm-level options, such as DNN-to-IMC mapping schemes, types of DNN algorithms, and using multiple cells for representing a higher-precision weight.

The WS-IMC mapping scheme, DNN-like algorithm, and linear quantization shows more robust immunity against variation. Although higher weight precision of S-MLC improves the baseline accuracy, it is also more susceptible to variation when the variation is high and the dynamic range is low. Multiple cells per weight and fine-tuning are two effective approaches to suppress inference accuracy loss if device-level solutions for reducing variation are not available. As for the PPA co-optimization, we found that memory devices with a large number of analog states spanning in a wide dynamic range do not necessarily lead to better IMC design. Low-bit MLC or even binary memory technology with G_H_/G_L_ < 10 and low variability, e.g. binary MRAM^25^ and FTJ^23^ with low conductance, deserves more attention.

## Methods

### Network structure

VGG-9 network for CIFAR-10 classification consists of 6 convolutional layers and 3 fully connected classifier layers. Image is processed through the stack of convolutional layers and 3 × 3 filters with a stride of 1. Max-pooling is performed over a 2 × 2 window and follow every 2 convolutional layers. Batch normalization and hard tanh as activation function are applied to the output of each convolutional layer. The width of convolutional layers starts from 128 in the first layer and increasing by a factor of 2 after each max-pooling layer. For the positive only IN in N-IMC and WS-IMC, the output of hard tanh activation function is scaled and normalized between 0 and 1.

VGG-16 network for Tiny ImageNet classification consist of 13 convolutional layers and 3 fully connect layer. Max-pooling is performed over a 2 × 2 window and follow every 2 or 3 convolutional layers. The width of convolutional layers starts from 64 in the first layer and increasing by a factor of 2 after each max-pooling layer.

### Quantized neural network training

We use quantize weights and activation to perform VMM calculation at run-time and compute parameter gradients at train-time, while the real-valued gradients of the weight are accumulated in real-value variable. Real-value weights are required for optimizer to work at all. The quantized weights and activations are transformed from the real-value variable by using the following deterministic linear quantization function:

$${x}_{Q}=LinQ\left({x}_{r},bitwidth\right)=Clip\left(\frac{{x}_{r}}{bitwidth},min,max\right),$$

and logarithmic quantization function

$${x}_{Q}=LogQ\left({x}_{r},bitwidth\right)=Clip\left(sign\left({x}_{r}\right)\times {2}^{round(log2|{x}_{r}|)},min,max\right),$$

where *x*_r_ is the original real-value variable, *x*_Q_ is the value after quantization, bitwidth is quantization bit precision, and *min* and *max* are the minimum and maximum scale range, respectively^24^.

The real-valued weight gets updated iteratively by using adaptive moment estimation (ADAM) optimization algorithm using the learning rate decayed by half every 20 or 40 epochs. Both CIFAR-10 and Tiny ImageNet datasets are trained over 200 epochs with a batch size of 256. All the results are obtained with PyTorch, a popular machine learning framework, and trained on an NVIDIA Tesla P100 GPU.

## Supplementary Information

Supplementary Information.
