# Supplementary material for: Device quantization policy in variation-aware in-memory computing design
Source: Sci Rep. 2022 Jan 7;12:112. doi: 10.1038/s41598-021-04159-x (PMC8741899; doi:10.1038/s41598-021-04159-x)
Supplement: Supplementary file 1 — Supplementary Information. [file 41598_2021_4159_MOESM1_ESM.pdf]

Supporting Information

Device Quantization Policy Strategy in Variation-Aware In-Memory Computing Design

S1. Effect of G-dependent σ

To evaluate the impact of G-dependent σ on inference accuracy, we assume a G-dependent σ model where the σ values of all the states except for the highest conductance state are the same and it is two times larger than the σ value of the highest conductance state and that used in the G-independent σ model. Figure S2 shows the comparison between the G-independent σ model and G-dependent σ model. For a larger dynamic range, the inference accuracy of the G-dependent σ model shows only a slight degradation compared to the G-independent σ model, suggesting that the noise induced by variation is dominated by the highest conductance state. This effect is visible in the linear-scale plot of Figure 4. However, for a smaller dynamic range, the noise is no longer completely dominated by the highest conductance state. The inference accuracy degrades due to the larger variation in the lower conductance states.

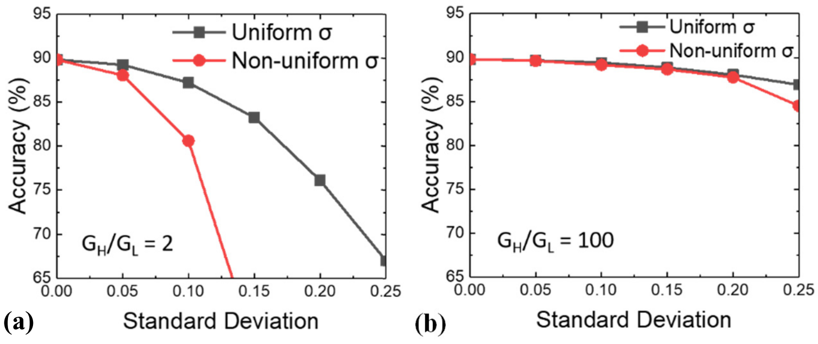

Figure S1. Impact of device variation on CIFAR-10 inference accuracy. Devices with uniform & non-uniform σ values are compared using the VGG-9 network. S-MLC, 2-bit weight, and WS-IMC are used. (a)G_H/G_L=2 and (b)G_H/G_L=100 are assumed.

S2. DNN-to-IMC Mapping – Comparison of N-IMC, C-IMC, and WS-IMC

Different mapping schemes show different immunity against weight variation. Figure S1 compares the inference accuracy of CIFAR10 dataset using a VGG-9 network with three different mapping schemes. 1-bit weight, 1-bit activation with varying dynamic ranges (G_H/G_L) were used in simulation. As σ increases, the inference accuracy degrades in all three schemes. N-IMC is the worst mainly due to the error accumulation from +*w* with broader distributions of G_L states as compared with − *w*. C-IMC shows improvement on inference accuracy compared with N-IMC because of the error cancellation originated from the complementary input. WS-IMC has the best immunity against weight variation due to its symmetric and tighter weight distribution using two adjacent memory cells rather than one.

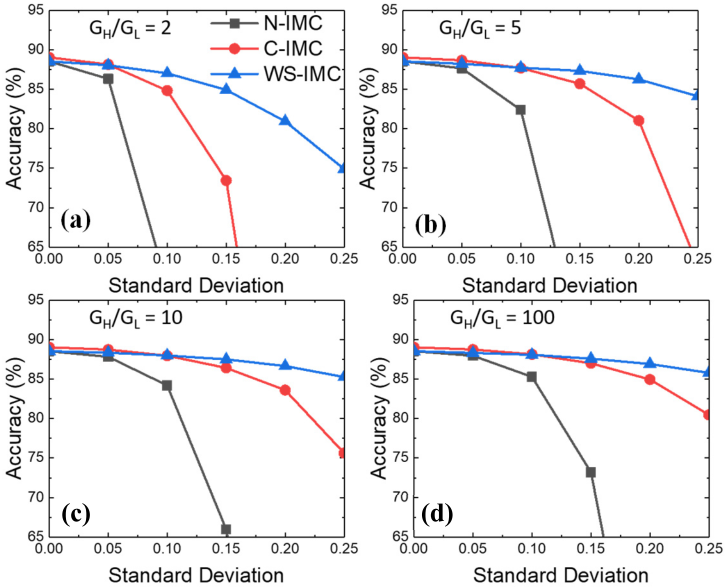

Figure S2. Influence of IMC mapping schemes. CIFAR-10 inference accuracy using VGG-9, 1-bit weight, 1-bit activation, (a) G_H/G_L=2 (b) G_H/G_L=5 (c) G_H/G_L=10 (a) G_H/G_L=100 are compared.

S3. Accurate Inference – Network Choice

Variation immunity is known to be sensitive to the choice of DNN algorithms. Figure S2 shows the comparison of variation immunity between VGG-16 & ResNet-18 by using the Tiny ImageNet dataset. Both networks show similar baseline accuracy in the ideal case with no variation. However, ResNet-18 shows worse variation immunity than VGG-16 regardless of the weight precision and dynamic range (G_H/G_L). Therefore, only the VGG-based DNNs with better immunity against variation are further evaluated in this study.

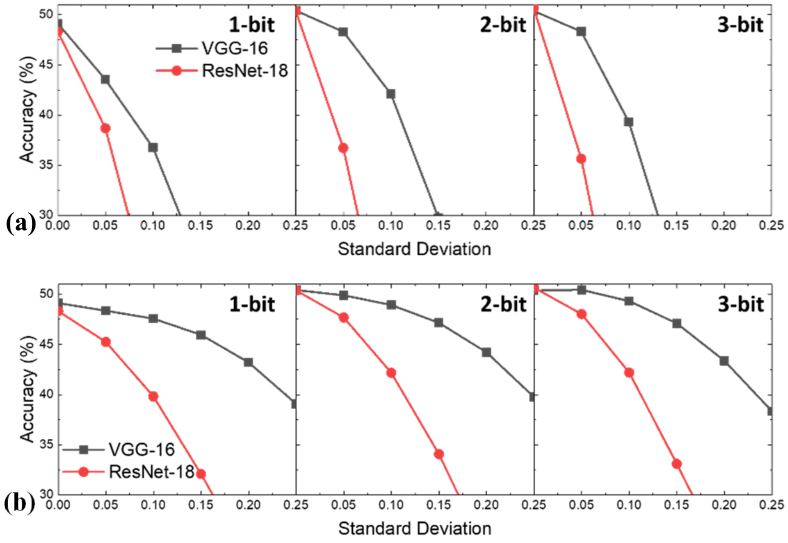

Figure S3. Variation-tolerant network. VGG-16 shows better variation tolerance in the Tiny ImageNet inference tasks than ResNet-18. S-MLC with 3-bit weight, WS-IMC, and (a)G_H/G_L=2 (b)G_H/G_L=100 is assumed.

S4. Additional Variation-Aware Strategies for PPA Co-Optimization

Here two kinds of variation-aware strategies are introduced to further improve variation immunity of DNN models: increasing the convolutional layers' channel size by two times and using higher activation precision of three bits. Both effectively improve variation immunity. However, the substantial penalties on area and energy restrict them only competitive in specific conditions, especially when σ is large. Figure S3 shows the area and energy estimation with these variation-aware strategies for a high-variation condition (σ=0.25). Different mapping schemes (S-MLC, D-MLC, and A-MLC) and dynamic ranges (2, 5, 10, and 100) are compared. The lowest

weight precision required to meet the accuracy target (88% CIFAR-10 inference accuracy; VGG-9) is indicated.

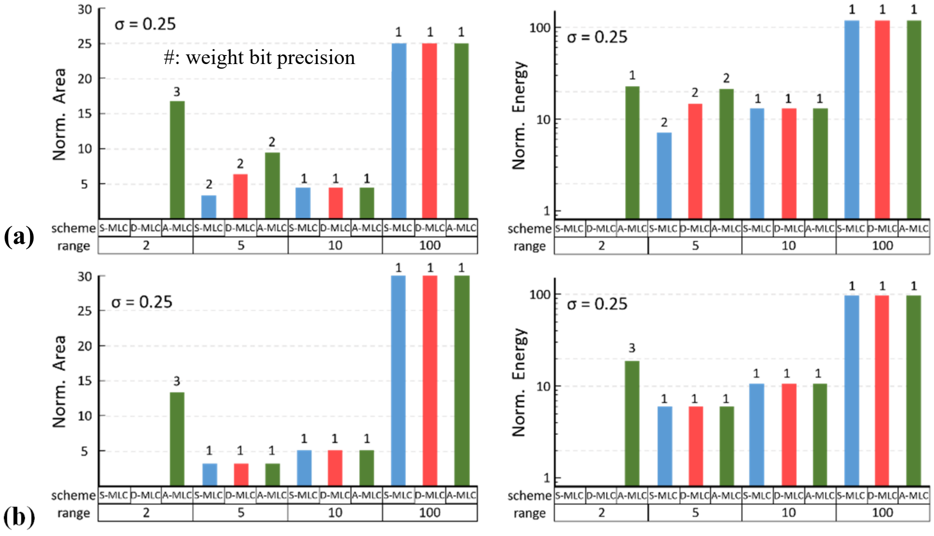

Figure S4. Area and energy estimation of IMC designs with either (a)wider channel or (b)3-bit activation. The improvements only exist in specific conditions with high σ.
